# Multiple Cranial Neuropathies in a Patient with Diffuse Large B-cell Lymphoma: Case Report and Review of Literature

**DOI:** 10.7759/cureus.2186

**Published:** 2018-02-13

**Authors:** Nakul Katyal, Anant Wadhwa, Pradeep C Bollu

**Affiliations:** 1 Department of Neurology, University of Missouri, Columbia, Missouri

**Keywords:** cranial nerve palsy, b cell lymphoma, central nervous system

## Abstract

Neuropathies can occur in patients with diffuse large B-cell lymphoma (DLBCL) at any stage of the disease as a presenting symptom or during later stages of illness. A wide spectrum of neurological association is known to occur with DLBCL, ranging from cranial nerve palsies to peripheral neuropathies. Evaluation of cranial and peripheral neuropathies in patients with DLBCL requires meticulous clinical, imaging, and electrodiagnostic testing. A 75-year-old right-handed female with a known history of multiple cranial neuropathies and DLBCL presented with acute dysphagia and upper extremity weakness of one-week duration. On evaluation, she was found to have right vagal nerve palsy. Cerebrospinal fluid (CSF) analysis along with flow cytometry testing showed CD19 and CD20 positive B cells, confirming neoplastic infiltration of CSF. We describe the case and review the literature of the association of cranial nerve palsies with DLBCL.

## Introduction

Cranial nerve palsies occur infrequently in patients with diffuse large B-cell lymphoma (DLBCL) and are seen in only 5% of the DLBCL cases [1]. DLBCL can affect any part of neuraxis from the brain, leptomeninges, spinal cord to peripheral nerves [2]. The possible pathogenic mechanism of cranial nerve involvement in DLBCL includes hematogenous spread, direct infiltration, or paraneoplastic phenomenon. Lesions predominantly affect oculomotor and facial nerves and are most frequently caused by the meningeal spread of neoplastic cells [3]. Neuropathies can present as the initial presentation of lymphoma or during the course of the disease. We describe a patient who presented with acute dysphagia and upper extremity weakness having been diagnosed with sixth and seventh cranial nerve palsies just two weeks prior. She had a known history of DLBCL and was receiving chemotherapy at the time of presentation. Flow cytometry analysis of cerebrospinal fluid (CSF) confirmed central nervous system (CNS) spread of DLBCL.

## Case presentation

**Figure 1:**
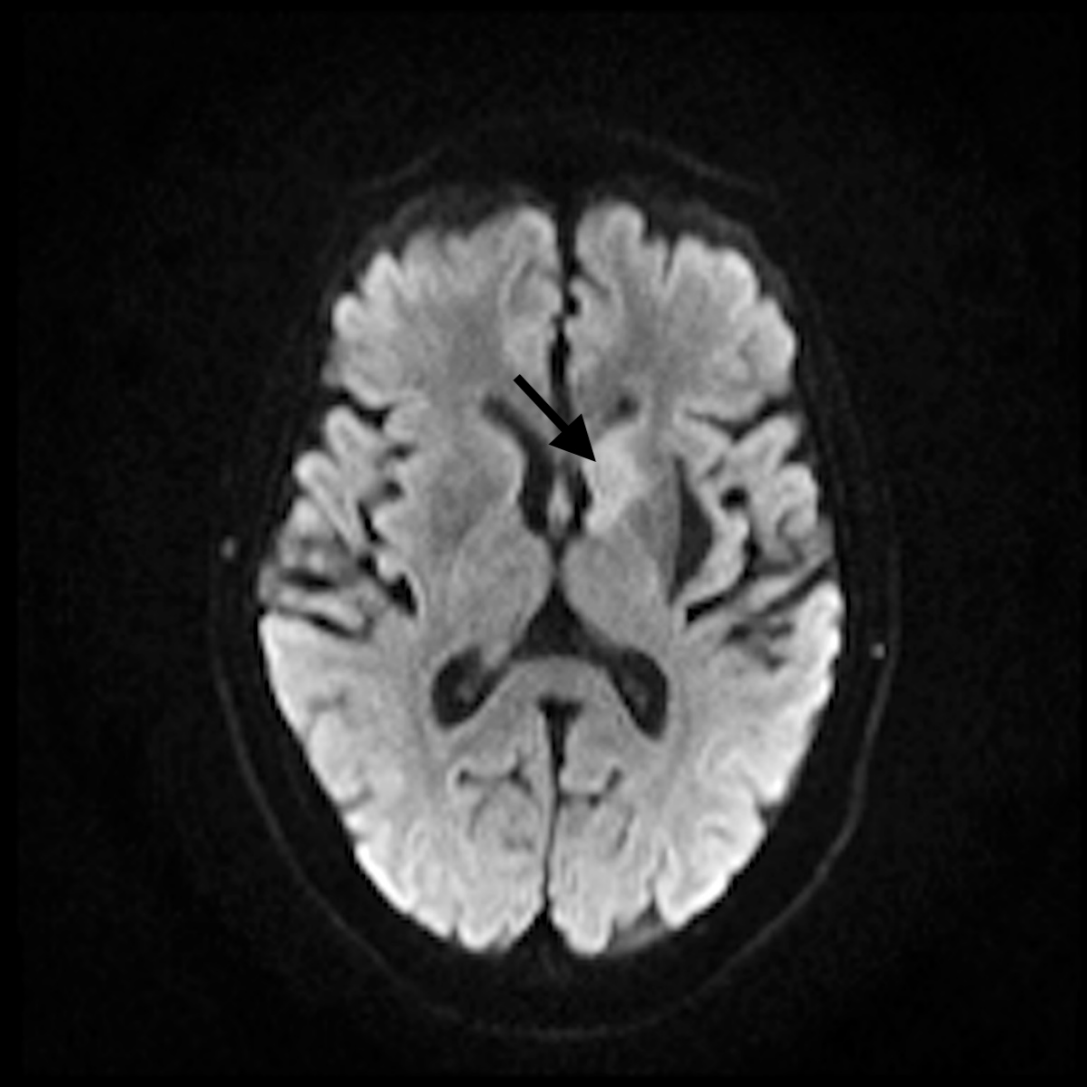
Magnetic resonance imaging (MRI) MRI showing increased signal intensity in the left caudate head on diffusion-weighted imaging (DWI).

**Figure 2:**
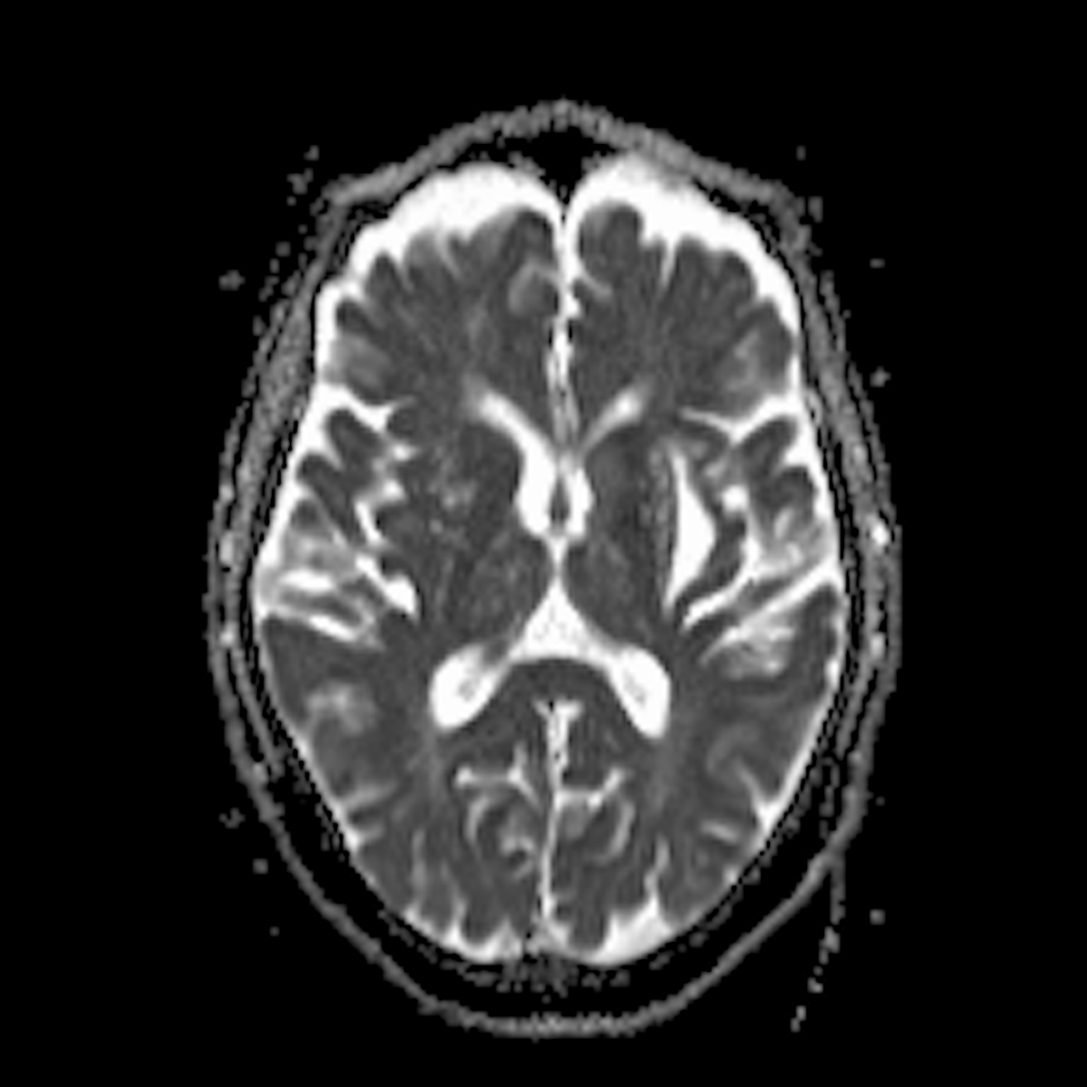
Magnetic resonance imaging (MRI) MRI without apparent diffusion coefficient (ADC) correlate.

**Figure 3:**
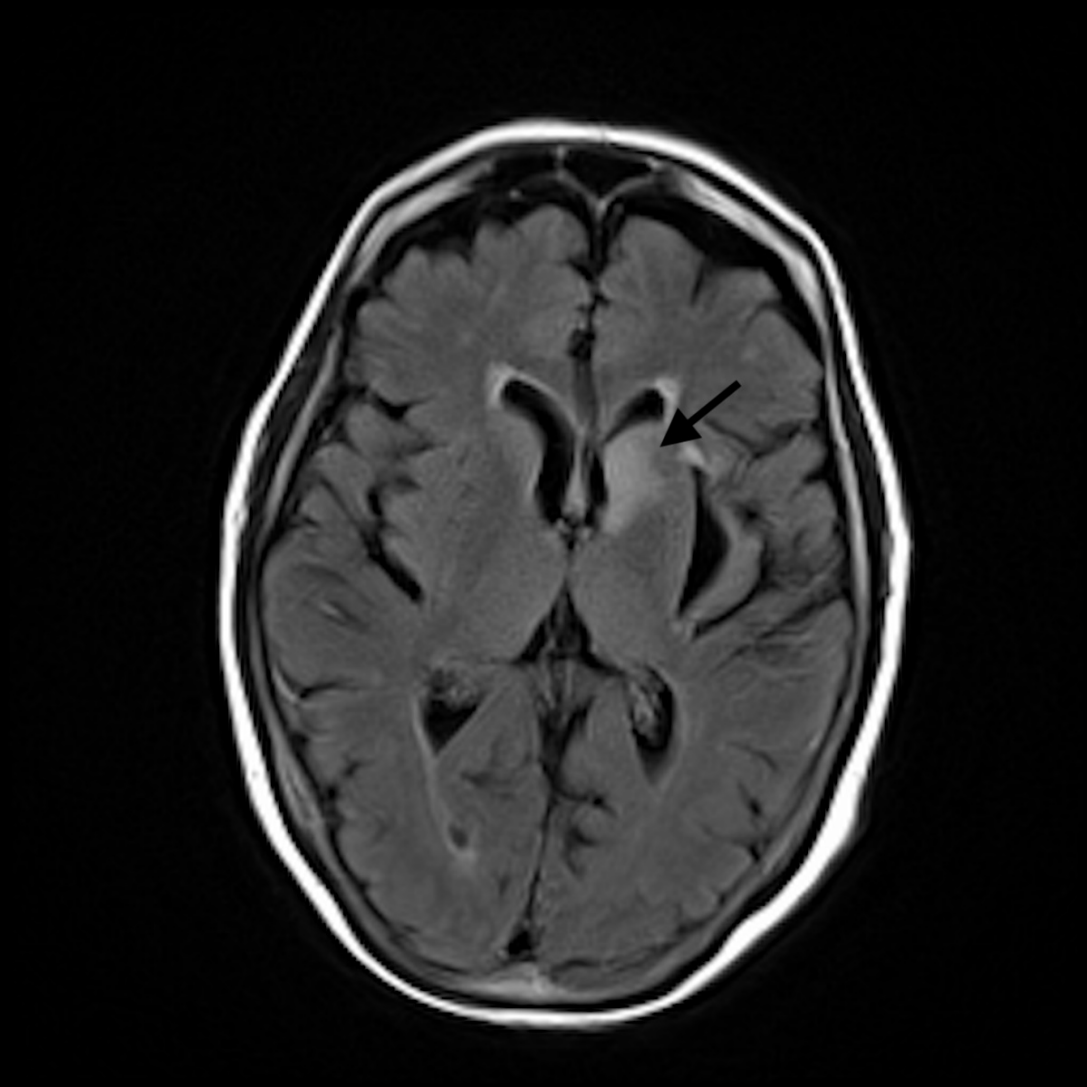
Magnetic resonance imaging (MRI) fluid-attenuated inversion recovery (FLAIR) imaging Increased signal intensity in the left caudate head on T2 FLAIR imaging.

A 75-year-old right-handed female was brought to the emergency department (ED) with complaints of dysphagia, increasing fatigue, upper extremity weakness more prominent on the left side, and upper extremity numbness for a one-week duration. She had a history of B-cell lymphoma and had received six cycles of chemotherapy (CHOP). Two weeks prior to presentation, she had presented with left-sided facial nerve palsy and left abducens nerve palsy. Magnetic resonance imaging (MRI) brain scan at that time had revealed a focus of increased diffusivity in the left caudate, with a hyperintense signal on diffusion-weighted imaging (DWI) without apparent diffusion coefficient (ADC) correlate. Her other medical comorbidities were congestive heart failure (ejection fraction <55%), depression, chronic headaches, history of pulmonary embolism, hypertension, hyperlipidemia, multiple transient ischemic attacks (TIA), and hyperhomocysteinemia. Her blood pressure (BP) at presentation was 160/83 mmHg. On examination, she had slurred speech with prominent nasal tone. Cranial nerve examination was significant for left abducens nerve palsy (restricted left eye abduction), lower motor neuron type of left facial weakness, and poor elevation of the right soft palate. Sensory examination showed decreased sensation on left medial forearm. Motor examination showed left-hand flexor and extensor weakness without loss of muscle bulk. MRI brain revealed an increase in signal intensity in the head of the left caudate nucleus compared to the scan from two weeks prior without any interval acute findings or contrast enhancement (Figures 1-3).

MRI C-spine did not reveal any myelopathy to explain left arm weakness although moderate-severe neural foraminal stenosis from C3-C6 was noted. Computed tomography (CT) of the chest and neck did not reveal any findings to explain dysphagia. Electromyography (EMG) and nerve conduction studies (NCS) showed changes consistent with chronic sensorimotor axonal polyneuropathy along with carpal tunnel syndrome and ulnar neuropathy on the left. Direct laryngoscopy confirmed right sided vocal cord paralysis consistent with right vagal nerve palsy. Lumbar puncture with CSF analysis showed abnormal cells consistent with malignancy. Flow cytometry analysis showed CD19 and CD20 positive B cells, thus confirming CNS involvement of DLBCL. Hematology/Oncology was consulted for CNS involvement of DLBCL. The patient however refused further chemotherapy and decided to proceed with palliative care. She was eventually discharged to a nursing facility.

## Discussion

Our case highlighted a rare presentation of multiple cranial neuropathies in a patient with DLBCL. We believe the underlying pathogenic mechanism was related to the meningeal spread of the malignant cells. DLBCL is the most common type of non-Hodgkin lymphoma (NHL) and accounts for 30% of all NHL cases. Neuropathies can occur in patients with DLBCL at any stage of disease, as a presenting symptom or during later stages of illness. A wide spectrum of neurological association is known to occur with DLBCL, ranging from cranial nerve palsies, paraneoplastic neuropathies, neuronopathies to peripheral neuropathies [4]. Peripheral lesions can occur at any level from nerve plexuses, nerve roots to peripheral nerves. Cranial nerve lesions most commonly present as oculomotor or facial nerve palsies. Neoplastic infiltration can occur outside the cranial vault where cranial nerves V-VIII forms a complex network of anastomosis [3]. Both anterograde and retrograde spread of lymphoma can occur at this site thus affecting multiple cranial nerves simultaneously. We believe the site of cranial nerve anastomosis outside the cranial vault was infiltrated by neoplastic cells at the time of initial presentation that resulted in left abducens and left facial nerve palsy and later in right vagal palsy in our patient. Lesions result from the meningeal spread of distant tumor in majority cases [3]. Leptomeningeal spread is the most common route of cranial nerve involvement in patients with DLBCL. However, a more aggressive form, primary CNS DLBCL can also cause cranial neuropathies in addition to widespread neoplastic infiltration of the brain, skull bone, orbit, nasal cavities, and cavernous sinuses [5]. The possible pathogenic mechanisms of nervous system involvement in DLBCL include direct infiltration of peripheral nerves, leptomeningeal spread, hematogenous spread, and immune complex-mediated damage. In patients receiving chemotherapy, neuropathy can happen as a result of damage caused by the chemotherapeutic agents such as vincristine. It typically results in a more generalized peripheral polyneuropathy [3]. The clinical triad of CNS involvement, cranial nerve lesions, and spinal/radicular symptoms/signs usually indicates leptomeningeal carcinomatosis [3,6].

Evaluation of cranial and peripheral neuropathies in patients with DLBCL requires conscientious clinical, imaging and electrodiagnostic testing. CSF analysis and flow cytometry testing are highly sensitive for diagnostic evaluation of DLBCL [1]. Neuroimaging is often the initial diagnostic modality used for investigating leptomeningeal and nerve plexus metastases. MRI with gadolinium enhancement can visualize leptomeningeal metastases which are typically linear or nodular in appearance [7]. Masses surrounding or infiltrating the nerve plexus can also be seen on MRI [8]. MRI brain showed increased signal intensity in the left caudate head on DWI and on T2 fluid-attenuated inversion recovery (FLAIR) imaging in our patient. Unilateral involvement of caudate nuclei may indicate the neoplastic invasion of the basal ganglia. Bilaterally symmetric diffuse abnormalities involving the lentiform and caudate nuclei typically results from systemic or metabolic causes, whereas asymmetric, focal, or discrete lesions affecting only part of the basal ganglia tend to indicate neoplastic infiltration [9]. NCS and EMG can aid greatly in anatomically localizing the lesions and distinguishing axonal and demyelinating neuropathies in patients with peripheral nerve involvement. Meningeal involvement should be investigated with CSF analysis and flow cytometry. Flow cytometry is highly sensitive in identifying neoplastic cells. With recent advances in immunotherapeutic, the prognosis for DLBCL has improved significantly over the last decade. Introduction of CD20 antibody (rituximab) has considerably improved outcome in patients with DLBCL. Mortality rates have been reduced to one third as compared to the pre-rituximab era. CHOP therapy (cyclophosphamide, doxorubicin, vincristine, and prednisone at 21-day intervals), however, still remains the standard combination for managing DLBCL [10].

## Conclusions

Cranial nerve involvement in patients with DLBCL results most commonly from leptomeningeal spread of neoplastic cells. Diagnosis requires clinical, imaging, cytological, and electrodiagnostic evaluation. Primary CNS lymphoma and cerebrovascular accidents present with similar clinical features. Recognizing and differentiating these conditions is essential, as treatment and prognosis varies significantly.
